# Neuroprotection by the noble gases argon and xenon as treatments for acquired brain injury: a preclinical systematic review and meta-analysis

**DOI:** 10.1016/j.bja.2022.04.016

**Published:** 2022-06-07

**Authors:** Min Liang, Fatin Ahmad, Robert Dickinson

**Affiliations:** 1 Anaesthetics, Pain Medicine, and Intensive Care Section, Department of Surgery and Cancer, Imperial College London, London, UK; 2 Royal British Legion Centre for Blast Injury Studies, Imperial College London, London, UK

**Keywords:** animal models, cardiac arrest, cardiopulmonary bypass, inert gases, ischaemic brain injury, ischaemic stroke, neuroprotection, traumatic brain injury

## Abstract

**Background:**

The noble gases argon and xenon are potential novel neuroprotective treatments for acquired brain injuries. Xenon has already undergone early-stage clinical trials in the treatment of ischaemic brain injuries, with mixed results. Argon has yet to progress to clinical trials as a treatment for brain injury. Here, we aim to synthesise the results of preclinical studies evaluating argon and xenon as neuroprotective therapies for brain injuries.

**Methods:**

After a systematic review of the MEDLINE and Embase databases, we carried out a pairwise and stratified meta-analysis. Heterogeneity was examined by subgroup analysis, funnel plot asymmetry, and Egger's regression.

**Results:**

A total of 32 studies were identified, 14 for argon and 18 for xenon, involving measurements from 1384 animals, including murine, rat, and porcine models. Brain injury models included ischaemic brain injury after cardiac arrest (CA), neurological injury after cardiopulmonary bypass (CPB), traumatic brain injury (TBI), and ischaemic stroke. Both argon and xenon had significant (*P*<0.001), positive neuroprotective effect sizes. The overall effect size for argon (CA, TBI, stroke) was 18.1% (95% confidence interval [CI], 8.1–28.1%), and for xenon (CA, TBI, stroke) was 34.1% (95% CI, 24.7–43.6%). Including the CPB model, only present for xenon, the xenon effect size (CPB, CA, TBI, stroke) was 27.4% (95% CI, 11.5–43.3%). Xenon, both with and without the CPB model, was significantly (*P*<0.001) more protective than argon.

**Conclusions:**

These findings provide evidence to support the use of xenon and argon as neuroprotective treatments for acquired brain injuries. Current evidence suggests that xenon is more efficacious than argon overall.

**Editor's key points**

• The noble gases argon and xenon are novel neuroprotectants that have been evaluated in preclinical studies, with variable results. Xenon (but not argon) has undergone early clinical trials.
• This systematic review and meta-analysis of the preclinical literature indicates that argon and xenon are neuroprotective. Xenon appears more effective than argon.
• These results encourage clinical trials of the use of xenon and argon in brain injury.

Acquired brain injuries (ABIs) are a major source of morbidity and mortality worldwide.^1^, ^2^, ^3^ ABI can be caused by either a traumatic injury (road accidents, accidental fall, sports injuries, violence) or ischaemic brain injury (ischaemic stroke, cerebral ischaemia secondary to cardiac arrest (CA), neurological injury after cardiopulmonary bypass (CBP), perinatal hypoxic–ischaemic encephalopathy). Individuals suffering from ABI, even mild head injuries or mild stroke, can exhibit a range of cognitive, motor, and emotional symptoms such as headaches, dizziness, fatigue, irritability, inattention, sleep disorders, memory deficit, nausea, anxiety, and depression.^4^, ^5^, ^6^, ^7^, ^8^ These symptoms can persist long term, severely impairing quality of life.^4^, ^5^, ^6^, ^7^, ^8^

At present, clinically proven therapeutic options are limited to thrombolysis for ischaemic stroke, cooling for out-of-hospital CA and perinatal hypoxic–ischaemic encephalopathy or non-specific interventions to stabilise physiology such as tissue oxygenation and intra-cranial pressure for traumatic brain injury (TBI).^9^^,^^10^ Effective pharmacologic interventions aimed specifically at preventing neuronal loss and improving outcome after injury have proved elusive and are urgently required. In the past 20 yr, after the discovery of their pharmacologic targets,^11^, ^12^, ^13^, ^14^, ^15^ interest has grown in the use of the noble gases xenon and argon as novel neuroprotectants to minimise or prevent the development of injury after ABIs.^16^, ^17^, ^18^, ^19^, ^20^

A number of *in vivo* studies with both noble gases have demonstrated efficacy as neuroprotectants in models of ABI.^17^^,^^19^^,^^21^ However, several studies have reported either no effect or minimal effect, or in some cases a detrimental effect.^22^, ^23^, ^24^ Given the contrasting findings in different animal models and in the same or similar models reported from different laboratories, a systematic review and meta-analysis is warranted to resolve the issue. Although there have been several narrative reviews and a few systematic reviews (without meta-analyses) of neuroprotection by xenon and argon, there has been only one systematic review and meta-analysis including both xenon and argon, by De Deken and colleagues,^25^ in 2016. This was limited to ischaemia–reperfusion injury and transplantation and included only four argon studies and 13 xenon studies on ischaemic brain injury in adult animals. Since the publication of the De Deken meta-analysis, several additional studies of the effects of argon and xenon on a variety of brain injury models have been carried out, including those examining their efficacy in TBI models. In this study, we aimed to conduct a systematic literature review and meta-analyses to evaluate the current evidence surrounding the neuroprotection of xenon and argon in adult animal models of ABI, in order to guide future preclinical and clinical studies.

## Methods

This systematic review and meta-analysis followed the Systematic Review Centre for Laboratory animal Experimentation (SYRCLE) and the Collaborative Approach to Meta-Analysis and Review of Animal Data from Experimental Studies (CAMARADES) guidelines.^26^, ^27^, ^28^ The study protocol was registered with the Open Science Foundation Registries (https://bit.ly/3pJzL2B).

### Literature search and study selection

The detailed search strategy including search terms used is shown in Supplementary Table S1. Searches were carried out on Ovid MEDLINE (PubMed, 1956 to 16 Nov 2021) and on Embase (1947 to November 16, 2021) databases. The reference lists of eligible literature were reviewed to identify any relevant papers missed in the search. In addition, we screened articles that had cited eligible papers.

### Inclusion criteria

Eligible studies were preclinical trials that explored the effects of noble gases in adult or juvenile animals exposed to brain injury, published in English. There were no restrictions on the year of publication, time of initiation, and duration of treatment, or concentration of therapeutic gas administration.

Specifically, we included articles that (1) studied neuroprotection in animals that received either xenon or argon treatment through spontaneous breathing or ventilator; (2) assessed (a) neurological function, (b) neuronal injury or lesion volume, or both; (3) had a control group that received identical treatments to the study group, whereby the only variation was in the gas treatment (in the studies that combined noble gas treatment with any other therapeutic regime, such as hypothermia, the control group was considered this treatment alone if such a group was included); and (4) with or without a sham group.

### Exclusion criteria

Studies were excluded if they (1) were human trials; (2) used neonatal animals; (3) did not investigate xenon or argon as neuroprotectants; (4) used a subarachnoid haemorrhage model (as this experimental model is very severe with high mortality); (5) lacked the necessary data for meta-analysis (e.g. group sizes not given), and these data could not be obtained from the authors; or (6) lacked the required outcome measures, for example only physiologic or inflammation parameters reported. We aimed to investigate the efficacy of argon and xenon as neuroprotectants; changes in inflammatory markers or number of microglia are complex to interpret in terms of neuroprotection (e.g. depending on activation state an increased number of microglia may be neuroprotective or neurotoxic). We therefore did not include any outcomes involving neuroinflammation in the meta-analysis.

### Implementation of literature search and screen

The literature search and screening were conducted independently by two reviewers (ML, FA). After a comprehensive search and removal of duplicates, title-based and an abstract-based screening was performed, followed by full-text review of potentially relevant studies against the inclusion criteria. Discrepancies of study selection or quality assessment between the reviewers were decided by a third researcher (RD).

### Quality assessment

The risk of bias for each included study was evaluated independently by two reviewers (ML, FA) using a modified version of the checklist developed by CAMARADES^29^^,^^30^ (see Supplementary material, Methods).

### Data extraction and transformation

Data were extracted independently by two reviewers (ML, FA). Discrepancies between the reviewers were identified by a third researcher (RD). If the discrepancy was not resolved via independent checking by the reviewers, the third researcher adjudicated. Most discrepancies were the former, but there were a few cases (e.g. counting the number of data points in a scatter plot) where the third researcher adjudicated. The dependent values were extracted from control group, treatment group, and sham group (if there was one). The parameters extracted were: (1) neurological evaluation, including motor, cognitive, and memory testing; (2) histologic evaluation, including infarct volume, neuronal count or density, number of dead or apoptotic neurones; and (3) body weight change. In animal brain injury studies, it is common that there is weight loss immediately after injury. The degree of weight loss is a measure of injury severity. We did not extract data that could not be unambiguously related to neuroprotection (e.g. physiologic data, quantification of neuroinflammation that may be helpful or harmful depending on context). For each outcome, the mean (*X*), standard error of the mean (sem), standard deviation (sd), and the total number of animals per group (*n*) were extracted. For animal experiments, a control group usually serves more than one treatment group. The number of treatment groups per control was therefore obtained from the original article, and the ‘true number of control animals ($n_{\text{c}}^{\text{′}})$’ was calculated using equation (1).^28^

(1) $$n_{\text{c}}^{\text{′}}=\frac{n_{\text{c}}}{\text{Treatment groups served by one control}}$$

For studies where numerical values of outcomes were not provided, data were extracted from calibrated digitised plots using a web-based plot digitiser tool (https://automeris.io/WebPlotDigitizer/). All raw data were transformed into a format compatible with the CAMARADES meta-analysis web-based application (see user guide, https://bit.ly/3EB4mFX). Additional information including the type of noble gas, species, injury model, initiation of treatment, duration of treatment, and general conclusions of the article were recorded.

We used normalised mean difference (NMD) as a measure of effect size because it allows outcomes measured on different scales (e.g. infarct volume and neurological deficit score) to be combined in the same meta-analysis (equation (2), where *x*_c_ is the mean value of control group, *x*_sham_ is the mean value of sham group, and *x*_rx_ is the mean value of treatment group).

(2) $$\text{NMD}=100\text{\%}\times \frac{\left({\bar{x}}_{\text{c}}-{\bar{x}}_{\text{sham}}\right)-\left({\bar{x}}_{\text{rx}}-{\bar{x}}_{\text{sham}}\right)}{\left({\bar{x}}_{\text{c}}-{\bar{x}}_{\text{sham}}\right)}$$

For more information, see Supplementary material, Methods.

### Meta-analysis

Pairwise meta-analysis and stratified meta-analysis were performed using the CAMARADES meta-analysis web application (https://camarades.shinyapps.io/meta-analysis-app/) or Stata (Version 16; StataCorp, College Station, TX, USA). Individual effect sizes were weighted using the inverse variance method, by the inverse of their squared standard error (1/se^2^).^28^^,^^30^ We performed the meta-analysis in two stages. First, for each of the included studies, we extracted the data from the paper as described above. For all the relevant outcomes included in each study a pairwise random effects model meta-analysis of NMDs between the control and treatment groups was performed using the inverse variance method for weight, the restricted maximum likelihood (REML) estimator for tau^2^ and the Q-profile method for the confidence interval (CI) of tau^2^ and tau. This gave a single overall effect size and se for each study.

These individual study effect sizes and se values were then included in the overall random effect meta-analyses for argon and xenon,^27^^,^^31^ using the inverse variance method for weight, the REML estimator for tau^2^ and the Q-profile method for the CI of tau^2^ and tau. The homogeneity of the therapeutic effects among all included studies was quantified using the heterogeneity index (*I*^2^) and tested using the *Q*-statistic with a nominal significance value of *P*<0.05.

Potential sources of heterogeneity were explored using stratified meta-analyses. The predefined potential sources of heterogeneity consisted of animal species, injury model, study quality, sample size calculation, randomisation, blinding to assessment of outcome, temperature control, and inclusion of sham group. The subgroup differences in stratified meta-analyses were tested with a χ^2^ test.

Between-study heterogeneity in the meta-analysis was examined by constructing funnel plots and Egger's regression.^32^ The influence of funnel plot asymmetry on summary effects was quantified using the trim-and-fill method.^33^

## Results

### Systematic literature review

**Fig 1:**
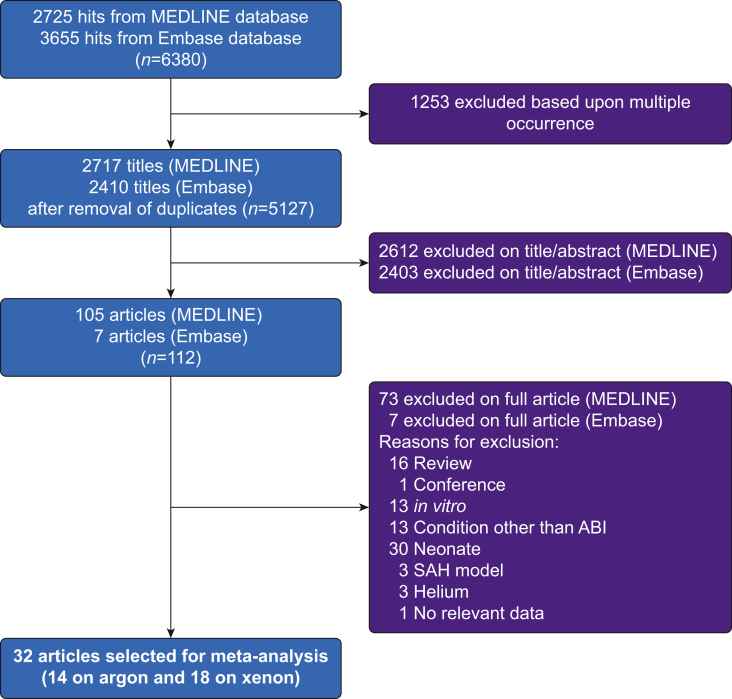
Results of systematic literature search strategy. Thirty-two articles were included in the meta-analysis. ABI, acquired brain injury; SAH, subarachnoid haemorrhage; no relevant data, study did not report relevant outcome measures (e.g. only physiological and inflammation parameters reported).

Our search strategy, shown in Figure 1, identified a total of 32 studies for meta-analysis, 14 for argon and 18 for xenon. The studies characteristics are summarised in Table 1. The experimental brain injury models identified were TBI, CA, CPB, and ischaemic stroke, and the species were mouse, rat, and pig. Overall, data were included from 1384 animals, of which 550 (228 mice, 42 pigs, and 280 rats) were from argon studies, and 834 (335 mice, 86 pigs, and 413 rats) were from xenon studies. The median study sizes (control, noble gas, sham) were 23 for argon and 31 for xenon. Of note, three studies involved more than 100 animals, one for argon^22^ and two for xenon.^38^^,^^61^

**Table 1:** Characteristics of included studies. BCCAO, bilateral common carotid artery occlusion; CA, cardiac arrest; CAE, cerebral air embolism; CCI, controlled cortical impact; CHI, closed head injury; CPB, cardiopulmonary bypass; CPR, cardiopulmonary resuscitation; MCAO, middle cerebral artery occlusion; MTH, mild therapeutic hypothermia; pMCAO, permanent MCAO; TBI, traumatic brain injury; tMCAO, transient MCAO; tPA, tissue plasminogen activator; ROSC, return of spontaneous circulation; VF, ventricular fibrillation.

First author, year	Species, strain, sex, age/weight	Trauma model	Treatment groups	Control group	Results with treatment	Treatment effect (%)	Standard error
Brücken, 2013^34^	Rats, Sprague–Dawley, male, 400–500 g	CA and CPR, 7 min of VF and ventilation stopping, 3 min of CPR	Argon 70% for 1 h, 1 h after successful CPR	70% N_2_/30% O_2_	Histopathologic and functional neurological outcome improved	21.65	3.995
Brücken, 2014^35^	Rats, Sprague–Dawley, male, 400–500 g	CA and CPR, 7 min of VF and ventilation stopping, 3 min of CPR	Argon 40% or 70% for 1 h, 1 h after successful CPR	70% N_2_/30% O_2_	Neurological impairment and neuronal damage index reduced	34.111	3.405
Brücken, 2015^36^	Rats, Sprague–Dawley, male, 400–500 g	CA and CPR, 7 min of VF and ventilation stopping, 3 min of CPR	Argon 70% for 1 h, 3 h or 1 h after successful CPR	70% N_2_/30% O_2_	Histopathologic and functional neurological outcome improved	39.938	4.097
Brücken, 2017^37^	Rats, Sprague–Dawley, male, 400–500 g	CA and CPR, 9 min of VF and ventilation stopping, 3 min of CPR	Argon 70% for 1 h + MTH (32–34°C) for 6 h, 1 h after successful CPR	70% N_2_/30% O_2_+MTH (32–34°C) for 6 h	Neurological impairment and neuronal damage index increased	–6.717	1.554
Campos-Pires, 2015^38^	Mice, C57BL/6N, male, 2.5 months old/24 (3) g	TBI: CCI, probe diameter 3 mm, impact velocity 8 m s^−1^, duration 150 ms, displacement 1 mm, craniotomy closed	Xenon 30%, 50% or 75% for 3 h; 15 min, 1 h, 3 h, or 6 h after injury	75% N_2_/25% O_2_	Neurological outcome and lesion volume improved	27.628	3.215
Campos-Pires, 2019^39^	Mice, C57BL/6N, male, 2.5 months old/23.9 (0.1) g	TBI: CCI, probe diameter 3 mm, impact velocity 8 m s^−1^, duration 150 ms, displacement 1 mm, craniotomy closed	Xenon 75% for 3 h, 15 min after CCI injury	75% N_2_/25% O_2_	Secondary injury reduced; short-term and long-term neurological outcome improved	49.469	8.931
Campos-Pires, 2020^40^	Rats, Sprague–Dawley, male, 13 weeks old/429 (7) g	TBI: CCI, probe diameter 4 mm, impact velocity 6 m s^−1^, duration 400 ms, displacement 3 mm, craniotomy closed	Xenon 50% for 3 h, 30 min after CCI injury	75% N_2_/25% O_2_	Functional outcome improved and neuronal loss reduced	62.482	6.735
Creed, 2020^22^	Mice, C57BL/6J, male, 8–10 weeks old	TBI: CHI, probe diameter 2 mm, impact velocity 6.8 (0.2) m s^−1^, displacement 3 mm, skull intact	Argon 70% or 79% for 24 h, 30 min after CHI injury	70% N_2_/30% O_2_, or 79% N_2_/21% O_2_	Functional neurological outcome and neuronal quantification did not improve	–2.412	2.142
David, 2003^41^	Rats, Sprague–Dawley, male, 280–300 g	MCAO, right internal carotid artery to middle cerebral artery, diameter 0.18 mm nylon with a distal cylinder (3 mm long and 0.38 mm diameter), removed 90 min later	Xenon 50% or 75% for 3 h, 15 min after MCAO period	Air	50% xenon, but not 75%, reduced infarct volume in cortex and striatum	31.425	20.693
David, 2008^42^	Rats, Sprague–Dawley, male, 250–280 g	MCAO, right internal carotid artery to middle cerebral artery, diameter 0.18 mm nylon with a distal cylinder (3 mm long and 0.38 mm diameter), removed 60 min later	Xenon 50% for 3 h, 2 or 3 h after MCAO	Medical air	Xenon given 2 h, but not 3 h, after MCAO reduced cortical volumes of infarction and improved behavioural outcomes	22.400	11.630
David, 2010^43^	Rats, Sprague–Dawley, male, 250–275 g	MCAO, right internal carotid artery, PE-10 catheter with a single clot measuring 40 mm in length, PE-10 catheter was removed 45 min later and tPA was administered	Xenon 37.5% or 50% or 75% for 45 min, during tPA injection; or xenon 50% for 3 h, after tPA injection	Medical air + tPA	(1) Xenon is a tPA inhibitor; (2) intra-ischaemic xenon dose dependently inhibits tPA-induced thrombolysis and subsequent reduction of ischaemic brain damage; (3) post-ischaemic xenon virtually suppresses ischaemic brain damage and tPA-induced brain haemorrhages and disruption of the blood–brain barrier	13.385	35.205
David, 2012^44^	Rats, Sprague–Dawley, male, 250–280 g	MCAO, middle cerebral artery, nylon thread, removed 60 min later	Argon 50% 1 h, 2 h after MCAO induction	Medical air	Cortical volumes of brain damage reduced, but subcortical brain damage increased and neurological outcome did not improve	1.771	8.141
Derwall, 2008^45^	Pigs, domestic (*Sus scrofa*), male, 3–4 months	CA and CPR, 8 min of VF and ventilation stopping, 6 min of CPR	(1) Xenon 70% for 1 h/5 h, 60 min after successful CPR; (2) xenon 70% for 1 h, 10 min after successful CPR	70% N_2_/30% O_2_	Xenon conferred functional neurological improvement even when treatment was delayed for 1 h, the early treatment with xenon translated to only marginal functional improvement	43.383	8.468
Fahlenkamp, 2014^46^	Rats, Sprague–Dawley, male, 250–295 g	MCAO, middle cerebral artery, intraluminal thread-occlusion technique for 2 h	Argon 50% for 1 h, 1 h after MCAO induction	50% N_2_/50% O_2_	Neuronal loss in ischaemic core reduced, but in the penumbra not reduced	29.289	36.043
Filev, 2021^47^	Rats, Wistar, male, 200–300 g	TBI, dosed contusion injury, a 50-g mass pin from a height of 10 cm, skull open	Xenon 70–75% for 1 h, 15–30 min after TBI induction	Air	Motor function improved	59.444	29.684
Fries, 2008^48^	Pigs, domestic (*Sus scrofa*), male, 3–4 months/32–39 kg	CA and CPR, 8 min of VF and ventilation stopping; 6 min of CPR	Xenon 70% for 1 h/5 h, 60 min after successful CPR	70% N_2_/30% O_2_	Histological outcomes, neurocognitive and neurologic function improved	49.810	6.115
Fries, 2009^49^	Pigs, domestic (*Sus scrofa*), male, 3–4 months/36.0 (2.6) kg	CA and CPR, 8 min of VF and ventilation stopping; 6 min of CPR	Xenon 70% for 1 h, 10 min after successful CPR	70% N_2_/30% O_2_	Neurological and histopathologic outcomes did not improve	3.291	11.429
Fries, 2012^50^	Pigs, domestic, male, 4 months/35.6 (2.0) kg	CA and CPR, 10 min of VF and ventilation stopping; 6 min of CPR	Xenon (70% for 1 h) + MTH (33°C for 16 h), 1 h after successful CPR	70% N_2_/30% O_2_+MTH (33°C) for 16 h	Histopathological and functional neurological outcome improved	13.402	3.553
Fumagalli, 2020^51^	Pigs, domestic, male, 39 (2) kg	CA and CPR, 12 min of VF and ventilation stopping; 5 min of CPR	Argon 50% or 70% for 4 h, after successful CPR	70% N_2_/30% O_2_	Neurologic recovery improved and brain injury ameliorated, with benefits are greater after 70% argon than 50% argon	33.837	11.492
Homi, 2003^52^	Mice, C57BL/6, male, 20–25 g	MCAO, right internal carotid artery to middle cerebral artery, a 6–0 nylon with a distal cylinder, removed 60 min later	Xenon 35% or 70% for 1 h 15 min, 15 min before MCAO induction	70% N_2_O/30% O_2_	Functional and histologic outcomes improved	22.029	4.451
Jungwirth, 2006^23^	Rats, Sprague–Dawley, male, 10 weeks/363 (17) g	CPB+CAE, 90 min of normothermic non-pulsatile CPB with flow rates of 160–180 ml min^−1^ kg^−1^, 10 equally sized CAEs (0.3 μl/single bolus) via the right internal carotid artery from 15 to 75 min of CPB	Xenon 56%, 20 min before CPB, during CPB, and 30 min after CPB	61% N_2_/34% O_2_/5% CO_2_	Neurologic dysfunction aggravated	–63.237	10.021
Jungwirth, 2011^24^	Rats, Sprague–Dawley, male, 10 weeks /315 (20) g	CPB+CAE, 90 min of normothermic non-pulsatile CPB with flow rates of 160–180 ml min^−1^ kg^−1^, 10 equally sized CAEs (0.3 μl/single bolus) via the right internal carotid artery from 15 to 75 min of CPB	Xenon 56% for 60 min before CPB with CAE/for 90 min during CPB with CAE/for 60 min after termination of CPB with CAE	61% N_2_/34% O_2_/5% CO_2_	Xenon administered immediately after (but not before or during) CPB and CAE impaired motor, cognitive, and histological outcome	–25.696	5.137
Limatola, 2010^53^	Mice, C57BL/6, male and female, 8 weeks /20–25 g	MCAO, right middle cerebral artery, a 6–0 nylon monofilament, removed 60 min later	Xenon 70% for 2 h, 24 h before MCAO induction	70% N_2_/30% O_2_	In both sexes, histologic and neurological functional outcome improved	39.631	3.219
Liu, 2019^54^	Rats, Wistar, male, 290–390 g	MCAO, left middle cerebral artery, a 4–0 nylon monofilament, removed 2 h later	Argon 50% for 1 h, 1 h after reperfusion	50% N_2_/50% O_2_	Neurological deficit and neuronal loss alleviated	21.721	4.624
Ma, 2003^55^	Rats, Sprague–Dawley, male, 12–14 weeks /350–380 g	CPB, 60 min of normothermic non-pulsatile CPB with flow rates of 160–180 ml min^−1^ kg^−1^.	Xenon 60% for 60 min, during CPB	65% N_2_/30% O_2_/5% CO_2_	Neurological and neurocognitive dysfunction improved	80.065	12.534
Ma, 2019^56^	Rats, Wistar, male, 10–12 weeks /250–300 g	(1) tMCAO, internal carotid artery to middle cerebral artery, nylon monofilaments with 0.38-mm diameter silicon tips, and removed 90 min later. (2) pMCAO, internal carotid artery to middle cerebral artery, nylon monofilaments with 0.27-mm diameter tips	(1) tMCAO: argon 70% for 24 h, immediately after reperfusion; (2) pMCAO: argon 70% for 24 h, immediately or 2 h after surgery	70% N_2_/30% O_2_	Neurological outcome, overall recovery, and infarct volumes improved	28.458	5.428
Metaxa, 2014^57^	Rats, Wistar, male, 2–3 months /270–320 g	BCCAO, both common carotids and doubly ligated	Xenon 50% for 45 min, 1 h after BCCAO	Air	Ischaemic neurones and the amount of volume loss in the cortex and hippocampus reduced	53.694	5.058
Moro, 2021^58^	Mice, C57BL/6J, male, 9 weeks	TBI: CCI, probe diameter 3 mm, impact velocity 5 m s^−1^, antero-posteriority –2.5 mm; laterality –2.5 mm, displacement 2 mm, craniotomy closed	Argon 70% for 24 h, 10 min after CCI	Air	Sensorimotor function, cognitive and structural outcome improved	17.873	2.822
Ristagno, 2014^59^	Pigs, domestic, male, 38 (1) kg	CA and CPR, 8 min of VF and ventilation stopping; 5 min of CPR	Argon 70% for 4 h, within 5 min after resuscitation	70% N_2_/30% O_2_	Neurological and histologic outcome improved	50.595	11.727
Ryang, 2011^60^	Rats, Sprague–Dawley, male, 250–295 g	MCAO, left internal carotid artery to middle cerebral artery, 3–0 monofilament nylon suture of 5 cm length, removed 2 h later	Argon 50% 1 h, 1 h after induction of tMCAO	50% N_2_/50% O_2_	Infarct volumes and composite adverse outcomes reduced	15.983	3.351
Sheng, 2012^61^	Rats, Wistar, male, 10–12 weeks /250–300 g	MCAO, right internal carotid artery to middle cerebral artery, diameter 0.25 mm nylon monofilament, removed 70 min later	Xenon 15% or 30% or 45% for 8 h, 20 h, 44 h, 90 min after reperfusion	70% N_2_/30% O_2_	Infarct size reduced, neurological outcome measures improved	18.255	1.896
Zuercher, 2016^62^	Rats, Wistar, male, 9–10 weeks	CA and CPR, 8 min of CA initiated with a mixture of potassium and esmolol, 8 min of CPR	Argon 50% for 24 h, 15 min after ROSC	50% N_2_/50% O_2_	Histologic or clinical outcome did not improve	–16.911	9.500

### Assessment of study quality

After assessment with the modified CAMARADES risk-of-bias checklist, 24 studies (75%) were high-quality low risk of bias (scores 7–9), whereas 8 (25%) of the studies were identified as moderate quality, moderate risk of bias (scores 4–6). No low-quality, high risk of bias (scores 1–3) studies were identified (Supplementary Table S2).

### Meta-analysis

#### Argon is neuroprotective

**Fig 2:**
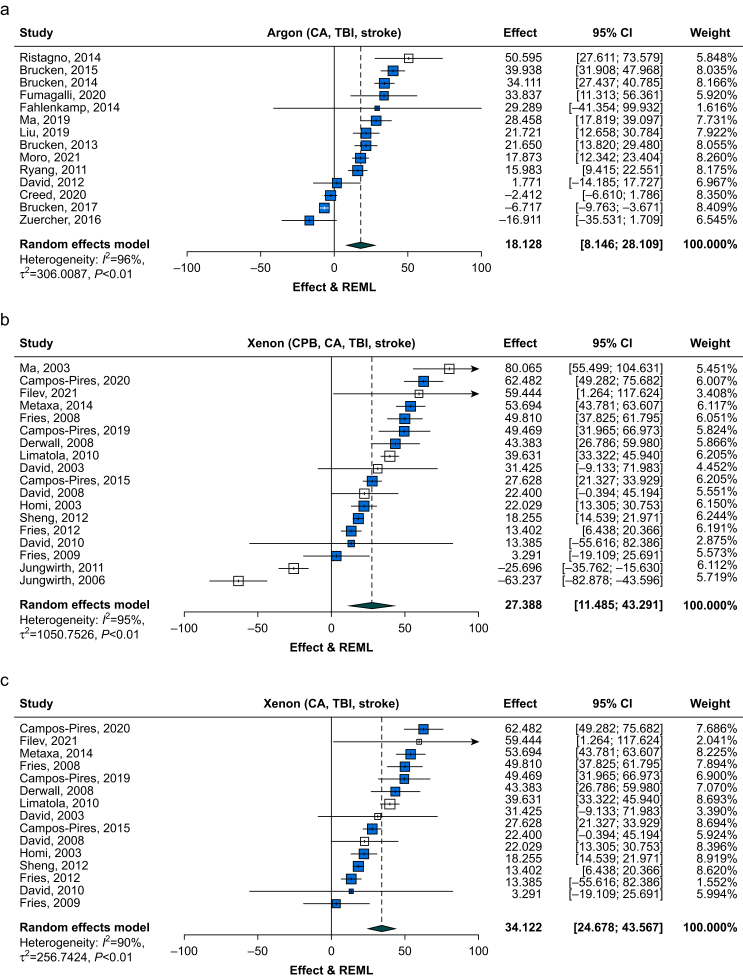
Forest plots comparing estimates of improvements in neurological outcome (effect size, confidence interval [CI], and weight) for: (a) argon including cardiac arrest (CA), traumatic brain injury (TBI) and stroke models; (b) xenon including cardiopulmonary bypass (CPB), CA, TBI, and stroke models; (c) xenon including only CA, TBI, and stroke models. Studies are ranked according to effect size. The size of each box is proportional to the study's weight in the meta-analysis with 95% CIs represented by horizontal lines. The box colour corresponds to study quality; high quality study with low risk of bias (blue) and medium quality study with medium risk of bias (white). The overall effect size from the meta-analysis random effects model is plotted as the green diamond, the width of which represents the 95% CI. A vertical dashed line denotes the overall mean effect, whereas a vertical solid line represents no (0%) effect. The first author and date of publication are listed on the left-hand column, whereas the right-hand column lists the effect size, CI, and weighting for each study.

In total, 14 studies examined the neuroprotective effects of argon. Argon was found to reduce neurological injury (combined histologic and neurological deficits) by 18.1% (95% CI, 8.1–28.1%; *Z*=3.6; *P*<0.001) with heterogeneity estimates (*I*^2^=96%, τ=17.5, *Q*=312) (Fig 2a).

Sources of heterogeneity were explored using stratified meta-analysis. Several differences in study design were identified among the included studies, including species, type of injury model, and study methods (e.g. randomisation, blinding, sample size calculation, and presence of sham group). These differences are potential sources of experimental or methodologic heterogeneity.

**Fig 3:**
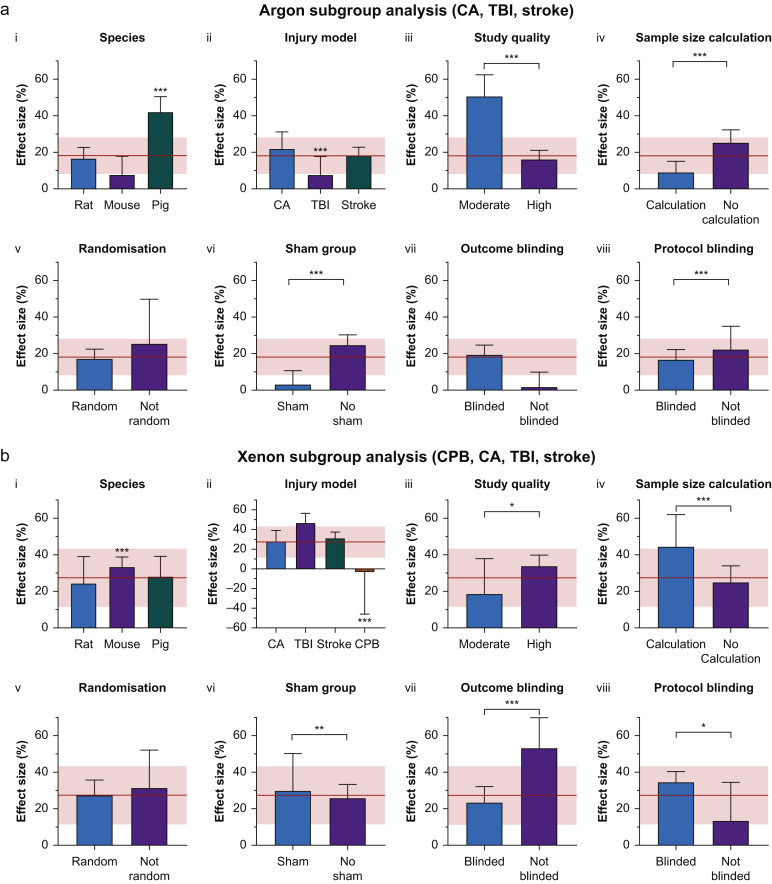
Neurological outcome effect size comparisons for subgroups in: (a) argon analysis cardiac arrest (CA), traumatic brain injury (TBI), and stroke models; and (b) xenon analysis, cardiopulmonary bypass (CPB), CA, TBI, and stroke models. (i) Species, rat (blue bar), mouse (purple bar) and pig (green bar). (ii) Brain injury models, CA (blue bar), traumatic brain injury (purple bar), stroke (green bar), and CPB, xenon only (brown bar). (iii) Study quality, moderate quality study (scores 4–6) (blue bar) *vs* high quality study (scores 7–9) (purple bar). (iv) Sample size calculation (blue bar) *vs* no sample size calculation (purple bar). (v) Randomisation (blue bar) *vs* no randomisation (purple bar). (vi) Sham group (blue bar) *vs* no sham group (purple bar). (vii) Outcome assessment blinded (blue bar) vs outcome assessment not blinded (purple bar). (viii) Injury protocol blinded (blue bar) *vs* injury protocol not blinded (purple bar). Bars are effect size, error bars represent standard error (se). Differences between subgroups were tested with χ^2^ test (^∗^*P*<0.05; ^∗∗^*P*<0.01; ^∗∗∗^*P*<0.001). The overall meta-analysis estimate and 95% confidence interval [CI] are indicated by the solid red line and the pink shading, respectively.

Subgroup analysis of results from stratified meta-analysis are shown in Figure 3a. Data are presented as effect size (se). Animal species, injury model, study quality, sample size calculations, presence of sham group, and blinding of injury protocol had a significant influence on the effect size in their respective comparisons (Fig. 3a-i, a-ii, a-iii, a-iv, a-vi, and a-viii; *P*<0.001). The effect size for pig models, 42.1% (8.4%), was larger than that for mouse or rat models, with a low heterogeneity (*I*^2^=4%). Effect size was smaller in the TBI injury model 7.6% (10.1%) compared with CA 21.9% (9.2%) and stroke 18.3% (4.5%). Moderate study quality was associated with a larger effect size, 50.6% (11.7%), although it should be noted there was only one moderate-quality study. No sample size calculation was associated with a larger effect size, 25.3% (7.0%). Lack of a sham group was associated with larger effect size 24.7% (5.5%), as was lack of injury protocol blinding 22.3% (12.6%). Lack of randomisation was associated with a greater effect size, but the difference was not significant (*P*>0.1). Blinding of outcome assessment was associated with greater effect size, but this was not significant (*P*>0.4). It should be noted that some subgroup comparisons have low power because of the low numbers of relevant studies (Supplementary Table S2). Nevertheless, where significant effects were observed in parameters related to study quality, as expected, the observed effect sizes were more conservative in higher quality studies and with parameters related to higher quality studies.

**Fig 4:**
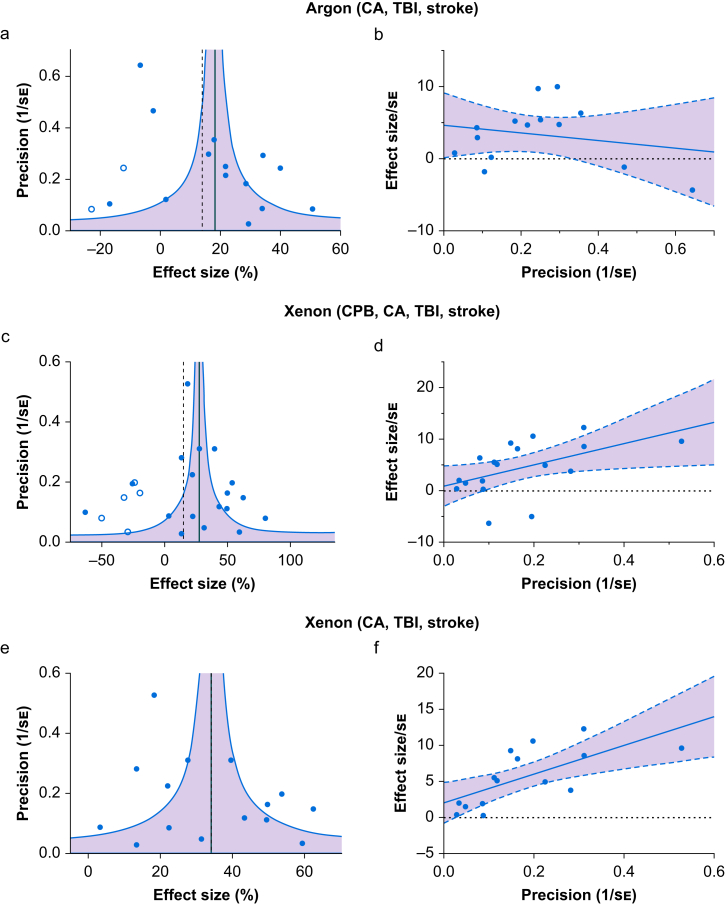
Heterogeneity analysis of the neuroprotection studies. (a) Funnel plot for argon including cardiac arrest (CA), traumatic brain injury (TBI), and stroke models. Trim-and-fill analysis detected asymmetry and two imputed studies (open circles) were suggested. (b) Egger's regression analysis for argon studies argon including CA, TBI, and stroke models. The line is the central estimate and the shading represents the 95% CI. The y-axis intercept of 4.7 (2.1) was significantly (*P*<0.05) different to zero indicating asymmetry. (c) Funnel plot for xenon including cardiopulmonary bypass (CPB), CA, TBI, and stroke models. The trim-and-fill analysis detected asymmetry and five imputed studies (open circles) were suggested. (d) Egger's regression analysis of the xenon studies including CPB, CA, TBI, and stroke models. The y-axis intercept of 0.89 (1.85), was not significantly (*P*=0.63) different to zero indicating a failure to detect asymmetry. (e) Funnel plot for xenon including only CA, TBI and stroke models. No asymmetry was detected by trim-and-fill analysis. (f) Egger's regression analysis of the xenon studies including only CA, TBI, and stroke models. The y-axis intercept of 2.0 (1.3) was not significantly (*P*=0.14) different to zero indicating a failure to detect asymmetry. Values are regression coefficients (standard error [se]). Study effect size in funnel plots are plotted on the x-axis, the reciprocal of the standard error, as a measure of study precision, is plotted on the y-axis. Vertical solid line represents the meta-analysis summary effect sizes and dashed vertical lines represent estimates including imputed studies, where present. Shaded area within curved lines in represents 95% confidence interval (CI) for the random-effects model.

A funnel plot asymmetry was identified using trim-and-fill analysis and two imputed studies were suggested on the left side, as shown in Figure 4a. The estimated effect size including the imputed studies was 13.9% (95% CI, 3.4–24.4%; *I*^2^=96%; *P*<0.01), a 4.2% reduction compared with the originally observed value, 18.1%. Heterogeneity was also evident in Egger's regression analysis where the intercept was positive and significantly different to zero (intercept=4.7 [2.0]; *P*<0.05) (Fig 4b).

### Xenon is neuroprotective

In total, 18 studies examined the effects of xenon. As shown in Figure 2b, xenon reduced neurological injury (combined histologic and neurological deficits) by 27.4% (95% CI, 11.5–43.3%; *Z*=3.4; *P*<0.001) with heterogeneity of the estimates (*I*^*2*^=95%, τ=32, *Q*=336).

Sources of heterogeneity were explored using stratified meta-analysis. The subgroup results are shown in Figure 3b (data presented as effect size [se]). Several aspects of study design were identified as having a significant effect on effect size (Fig. 3b-i, b-ii, b-iii, b-iv, b-vi, b-vii, and b-viii) including species (*P*<0.001), type of injury model (*P*<0.001), study quality (*P*<0.05), sample size calculation (*P*<0.001), presence of sham group (*P*<0.01), blinding of outcome measurement (*P*<0.001), and blinding of injury protocol (*P*<0.05).

Studies using mice had a larger effect size, 33.3% (5.5%) (Fig. 3b-i). CPB models were associated with a qualitatively different, negative, mean effect size of –3.3% (42.6%), in contrast to the positive effect seen for other injury models (Fig 3b-ii) (TBI: 46.8% [9.7%], stroke: 31.0% [6.2%]; and CA: 28.0% [11.1%]). Moderate study quality (Fig 3b-iii) was associated with a smaller effect size of 18.7% (19.1%). Studies with sample size calculation and inclusion of a sham group had larger effect sizes (Fig 3b-iv and b-vi). Studies with unblinded outcome assessment had larger effect sizes than those of studies in which the outcomes were assessed blinded (Fig 3b-vii). In contrast, studies with unblinded injury protocol had a smaller effect size (Fig 3b-viii). However, randomisation did not have a significant effect on the effect size (Fig. 3b-v). Lack of temperature control during treatment (data not shown) was associated with significantly (*P*<0.001) greater effect size, 37.0% (5.0%), compared with temperature control, 25.1% (10.0%).

Trim-and-fill analysis identified asymmetry in the funnel plot, and five imputed studies were suggested on the left-hand side, as shown in Figure 4c. The estimated effect size including the imputed studies was 15.0% (95% CI, –1.0%–31.0%; *I*^2^=98%; *P*<0.1), a 12.4% reduction compared with the originally observed value, 27.4%. In contrast, Egger's regression did not suggest the presence of asymmetry with the intercept not significantly different to zero, 0.89 (1.83) (*P*=0.63; Fig 4d).

Because we identified the effect of xenon in the CPB model as having a negative effect size (different sign and magnitude to the CA, TBI, and stroke models), we hypothesised that inclusion of the CPB model could explain some of the heterogeneity and the asymmetry detected by trim-and-fill analysis. In order to test this hypothesis, and to facilitate comparison with the argon studies that include CA, TBI, and stroke models, but not the CPB model, we carried out a sensitivity analysis by running the xenon meta-analysis without the CPB studies. Including the remaining 15 xenon studies in the meta-analysis (Fig 2c), xenon reduced neurological injury by 34.1% (95% CI, 24.7–43.6%; *Z*=7.1; *P*<0.0001) with heterogeneity of the estimates (*I*^2^=90%, τ=16, *Q*=138). We conducted a subgroup analysis of the xenon studies excluding CBP models (Supplementary Fig. S1). Significant effects on the effect size were observed for animal species (*P*<0.05), injury model (*P*<0.01), study quality (*P*<0.001), sample size calculations (*P*<0.05), presence of a sham group (*P*<0.01), and unblinded injury protocol (*P*<0.001). Lack of temperature control during treatment (data not shown) was associated with significantly (*P*<0.001) greater effect size, 37.0% (6.0%), compared with temperature control, 33.8% (5.0%).

Trim-and-fill analysis on xenon studies excluding CPB detected no asymmetry and no imputed studies were suggested, as shown in Figure 4e. Egger's regression analysis was consistent with this, detecting no asymmetry with an intercept not significantly different to zero (intercept=2.0 [1.3], *P*=0.14) (Fig 4f).

### Xenon is more neuroprotective than argon

**Fig 5:**
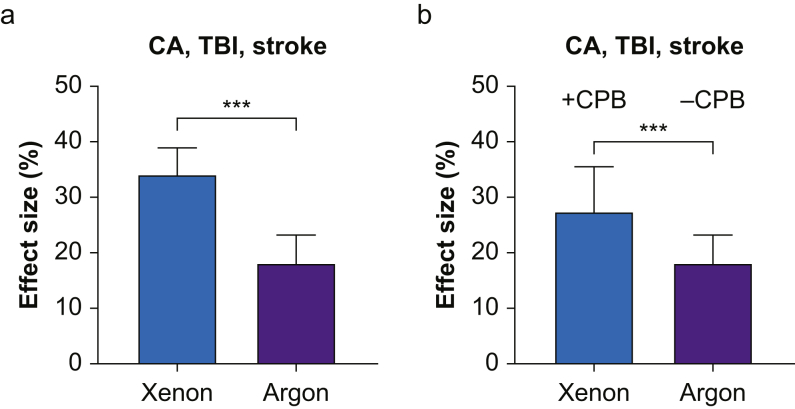
Neuroprotective effects of xenon and argon. (a) Comparison of overall neuroprotective effects of xenon (blue bar) and argon (purple bar) including cardiac arrest (CA), traumatic brain injury (TBI) and stroke models for both gases. (b) Comparison of overall neuroprotective effects of xenon (blue bar) and argon (purple bar), including cardiopulmonary bypass (CPB) model in xenon group only. Bars represent the effect size (%) and error bars represent standard error (se); ^∗∗∗^*P*<0.001, χ^2^ test.

Finally, we compared the efficacy of xenon and argon with a global stratified meta-analysis of all 32 studies with ‘gas treatment’ as a categorical variable. To make a comparison of xenon and argon on the same three models, we first compared only the CA, TBI, and stroke models (29 studies) as above. In this case, the effect of xenon increased to 34.1% (95% CI, 24.7–43.6%; se=4.8%) and the effect size was significantly (*P*<0.001) greater than that of argon, 18.1% (95% CI, 8.1–28.1%; se=5.1), as shown in Figure 5a. If we included all models including CPB in the xenon analysis, the xenon effect size reduced to 27.4% (95% CI, 11.5–43.3%; se=8.1), but was still significantly (*P*<0.001) greater than that of argon (Fig 5b).

## Discussion

### Systematic review

We identified 32 studies for inclusion in the meta-analysis, with publication dates from 2003 to 2021. All of the studies were of high (*n*=24) or medium quality (*n*=8) using a modified CAMARADES scoring. The CAMARADES checklist is a widely used risk of bias tool for preclinical studies and provides an objective scoring system aiming to assess internal and external validity aspects of study quality.^29^^,^^30^ The checklist was originally developed to assess preclinical models of ischaemic stroke,^29^^,^^30^ but has been used for models of other neurological conditions including TBI, cardiac arrest, Alzheimer's disease, and Parkinson's disease.^63^, ^64^, ^65^, ^66^, ^67^ There was one moderate-quality study in the argon group, that had the highest effect size of that group. There were seven moderate quality studies in the xenon group distributed equally on both sides of the summary effect estimate. Overall, as expected, higher quality studies were associated with greater precision (lower variance) in effect size estimate than lower quality studies, but there were exceptions. The moderate quality studies tended to have been published earlier, with all but one published between 2003 and 2014. The increase in number of higher quality studies in more recent years may reflect the more exploratory nature of the early studies and improvements in preclinical experimental design driven by funding body mandates on design and power calculations and the increasing costs of animal studies.

### Meta-analyses

The primary finding of the meta-analysis is that for both argon and xenon the summary effect size was positive with 95% CIs that do not include zero, indicating significant (*P*<0.001) neuroprotection and improved neurological outcomes for both gases.

### Argon

The overall summary effect size for argon was 18.1% (95% CI, 8.1–28.1%). Our finding of significant neuroprotection contrasts with an earlier meta-analysis of De Deken and colleagues^25^ that reported no significant protection by argon in ischaemic brain injury in rodents. The study by De Deken and colleagues was carried out in 2016 and included only four argon brain injury studies. Since the earlier work, an additional 10 argon studies have been published that are included in the 14 argon studies in our analysis. It is not straightforward to directly compare the effect sizes in our study with the results of De Deken and colleagues because these authors used the standardised effect size (SES) measure whereas we used the NMD measure of effect size. De Deken and colleagues reported SES values of 1.58 (95% CI, –1.31 to 4.47) and 2.31 (95% CI, –0.25 to 4.86) for histologic and neurological injury, respectively; the mean values are positive, consistent with a neuroprotective effect. However, the leftmost 95% CIs cross zero, leading the authors to state that they could not conclude if the difference in means indicated a significant neuroprotective effect. Our NMD effect size for argon including 10 additional studies was 18.1% (95% CI, 8.1–28.1%). The mean value is positive indicating a neuroprotective effect, but in our study the leftmost 95% CI is greater than zero, indicating a significant neuroprotective effect. We believe that our findings are consistent with the earlier work of De Deken and colleagues with both results having mean values indicating protective effect, but that the additional studies included in our work have increased the precision in the effect size estimate such that on the current evidence the neuroprotective effect of argon is significant.

Subgroup analysis identified pig models (two studies) and ‘moderate study quality’ (one study) as being associated with increases in effect size, with mean values outside the CI of the summary effect. TBI models (two studies) and inclusion of sham group (five studies) were associated with reduction in effect size with mean values outside the CI of the summary effect. Trim-and-fill analysis of the funnel plot suggested two imputed studies on the left of the plot (negative effect size) that would reduce the overall summary effect. Egger's regression also identified significant asymmetry in the positive direction consistent with the funnel plot.

### Xenon

The overall summary effect size for xenon was 27.4% (95% CI, 11.5–43.3%). The significant overall neuroprotection by xenon that we observed is consistent with the findings of De Deken and colleagues^25^ in ischaemic brain injury in rodents, although a quantitative comparison of effect sizes is not straightforward as that study used SES measure. Subgroup analysis identified the effect size for CPB models as qualitatively different with a negative sign indicating an overall detrimental effect of treatment, and the mean value was outside the 95% CI of the summary estimate. This suggests that xenon may not be beneficial in this indication. In addition, within the CPB studies there was heterogeneity in effect size and experimental protocols, with two models (negative effect size) including injection of air bubbles to induce air embolism,^23^^,^^24^ whereas a third study (positive effect size) did not include air embolism.^55^ The CPB models were only used in the xenon studies, were heterogeneous, and differed significantly from the other models (see below). We hypothesised that these studies would add to the heterogeneity in the overall xenon analysis. Trim-and-fill analysis of all the studies including CPB suggested five imputed studies on the left of the plot (negative effect size) that would reduce the overall summary effect. In contrast, Egger's regression did not find asymmetry and was not consistent with the trim-and-fill analysis. If the asymmetry suggested by the funnel plot was attributable to a true difference in effect size for the CPB model, then removing this model from the analysis should reduce asymmetry. Excluding the CPB models from the funnel plot analysis removed the asymmetry and no imputed studies. Egger's regression excluding CPB models did not detect any asymmetry. Taken together, these findings are consistent with our hypothesis that asymmetry was attributable to inclusion of CPB models in which xenon has a different effect. If we include only the CA, TBI, and stroke models in the xenon analysis, the estimated effect size of xenon increases to 34.1% (95% CI, 24.7–43.6%; *P*<0.0001).

### Heterogeneity

Meta-analysis identified heterogeneity in both argon and xenon studies with high heterogeneity indices (*I*^2^). The substantial heterogeneity observed (argon: *I*^2^=96%; xenon [CPB, CA, TBI, stroke]: *I*^2^=95%; xenon [CA, TBI, stroke]: *I*^2^=90%) is unlikely to be attributable to sampling errors or publication bias (‘missing studies’), but rather differences in the study methods themselves, and that the xenon studies included a different injury paradigm (CBP) not present in the argon studies. To increase generalisability of our findings, we included different species and different injury models that may involve different underlying pathophysiology. Given the differences inherent in comparing animal studies, high heterogeneity values are expected, and our values are similar to those observed in the earlier analysis by De Deken and colleagues.^25^ In preclinical research there are often substantial differences in the study design and outcome measures used in different studies. Although some aspects contributing to study heterogeneity such as lack of randomisation or blinding can be assessed with risk of bias checklists, or from other information on study methodology and experimental design, animal models are complex and have multiple sources of heterogeneity. There may be heterogeneity owing to different severities of injury and timing of the interventions in the same injury type. An additional factor is that in most animal models, for ethical and welfare reasons, the brain injury occurs under general anaesthesia, a situation that does not happen in most clinical scenarios (CPB and reperfusion after thrombectomy are exceptions). In addition, we grouped together behavioural and histologic outcomes in the meta-analysis. Even for similar outcomes, different scales are used by different laboratories. For example neurological outcomes were assessed using a ‘neurological deficit score’ in most studies but were examined using the ‘neurologic alertness score’ in two studies.^51^^,^^59^ The proportion of damaged neurones, which is typically presented as percentage loss or reduction in density, was graded into discrete levels in some articles to obtain a neuronal outcome score.^49^^,^^50^ Given the high heterogeneity, that is typical of similar preclinical studies, the summary estimates of the random-effects model are best interpreted as a summary of the included studies, rather than an expected effect size under specific well-characterised conditions.^28^

Another possible source of the heterogeneity is reporting bias. The most common form of this is publication bias, which is usually attributable to the preferential publishing of positive findings over neutral or negative results.^32^ The estimate of the argon and xenon effect sizes were suggested to be slightly enhanced according to trim-and-fill analysis, and two imputed studies were suggested for argon and five for xenon (including the CPB model). Egger's regression identified asymmetry in argon data consistent with the funnel plot. However, Egger's regression identified no asymmetry in the xenon group, suggesting no missing publications. In the case of xenon, the asymmetry in the funnel plot can be explained by the heterogeneity resulting from inclusion of a different injury model (CPB) that may have a true different effect. No funnel plot heterogeneity was observed when CPB models were not included in the meta-analysis. It is likely that the asymmetry identified in the argon studies by both trim-and-fill analysis and Egger's regression can be explained by methodologic heterogeneity between studies, rather than publication bias. However, we cannot rule out publication bias completely.^68^

### Subgroup analysis

In order to increase the generalisability of interpretation of the subgroup parameters, we will discuss the subgroup findings for the argon and xenon studies together. Subgroup analyses may provide a more accurate estimate of effect size for a specific condition (e.g. disease model). An important caveat is that a lack of a significant difference in effect size between subgroups does not necessarily prove that those subgroups result in equal effects. This is partly because animal studies are so varied (e.g. species, methodologies, study features), and that the information provided by the summary effect size is pooled.^27^ In addition, if the total number of studies is modest, subgroups may have few studies and the analysis may be underpowered to detect differences.^27^ Consequently, it can be more difficult to identify significant differences between subgroups.

Nevertheless, several noteworthy findings emerged from subgroup analyses of our pre-defined factors of species, injury, model, overall study quality, and individual components of study design. Significant differences were observed with different species in argon and xenon studies. Pig models in the argon studies have a higher mean effect size than rat or mouse models and they are outside the 95% CI of the overall estimate. In the xenon studies (including CPB), mouse models had an effect size larger than the summary estimate mean, but within the 95% CI of the summary estimate. When CPB models were not included, rat models had a higher effect size but was within the 95% CI of the summary estimate. Comparing the different injury models, cardiac arrest and stroke models were similar to the summary estimate for both argon and xenon studies. Interestingly, xenon appeared most effective in the TBI model (mean slightly above the higher 95% CI of summary estimate), whereas argon had the least beneficial effect in the TBI model (mean slightly below lower 95% CI of summary estimate). However, CBP (only present in the xenon studies) was associated with a qualitatively different, negative, effect size well outside the lower 95% CI of the summary estimate. Three studies investigated CPB, all using rats, but there was methodologic heterogeneity. Ma and colleagues^55^ reported a positive effect size of 80% indicating xenon improved outcome, whereas Jungwirth and colleagues^23^^,^^24^ had overall negative effect sizes of –26% and –63%, indicating detrimental effect on outcome. The studies that reported a detrimental effect of xenon used a model that incorporated addition of an air embolism after CPB, and it was proposed that xenon may augment the size of gas bubbles.^23^^,^^24^ However, experimental measurement of the effect of xenon on gas bubbles suggests that any size increase is modest (≤10% increase in diameter).^69^ Overall, the current preclinical evidence is equivocal but suggests that xenon may not be of benefit in CPB models. It should be noted that the model used by Jungwirth and colleagues^23^^,^^24^ involves deliberate injection of air emboli via carotid artery, and it is not clear how precisely this models the clinical scenario. Xenon has undergone a small clinical feasibility and safety study (*n*=16 patients) that reported xenon was both safe and feasible in CPB patients.^70^ Although this study did not include neurological outcomes, it did report a reduction in serum S100β (a biomarker of neuronal injury) levels in the xenon group.^70^ Of note, the xenon treatment protocol in the clinical study (before, during, and after CPB) was different to the animal treatment paradigms of Jungwirth and colleagues^23^^,^^24^ where xenon was administered either only before CPB, only during CPB or only after CPB. Whereas Jungwirth and colleagues 2006^23^ only used xenon after CPB, the later publication by the same authors compared the three different treatment paradigms.^24^ Interestingly in these animal studies, significant detrimental effects were reported only in the paradigm when xenon was given only after CPB.^23^^,^^24^ We believe that further studies (both preclinical and clinical) will be required to resolve whether xenon is beneficial in CPB. With respect to the animal models, important questions to resolve are:

(1) How well do the models with deliberate injection of air emboli model the clinical scenario?
(2) What is the appropriate time for initiation of xenon treatment to model the clinical scenario?

The clinical study of xenon in CPB^70^ addressed feasibility and safety, and was not designed to assess efficacy or neurological outcome. Xenon's efficacy and safety in a larger clinical CPB cohort remains to be addressed in further studies. Whether or not argon is beneficial in CPB has not yet been addressed by any preclinical or clinical study that we could identify.

Study quality was associated with significant difference in effect size in both argon and xenon studies, with high-quality studies being close to the overall summary estimates and having lower variance. Individual aspects of study quality, sample size calculation, presence of sham group, and outcome blinding were associated with significant difference in effect size in both studies. In the argon studies no sample size calculation was associated with a larger effect size, and in the xenon studies it was associated with a smaller effect size. It has been noted that many individual animal studies are underpowered and that this may result in a bias.^71^ Underpowered studies may only be able to detect larger effect sizes, and this can bias the overall results in either direction, either by favouring reporting of the larger positive effect sizes, or by failing to detect smaller positive effect sizes and erroneously reporting no effect. It was unsurprising that not blinding outcome assessment or injury protocol was associated with significantly different effect sizes in the xenon studies, with blinding associated with mean effect size close to the overall estimated effect size. In the xenon studies no blinding of outcome assessment was associated with an increased effect size, as might be expected. Temperature control during treatment in the xenon studies was associated with an effect size close to the overall effect size, as expected for a potential confounding parameter. All of the argon studies included temperature control during treatment. Hypothermia improves neurological recovery in animal models,^72^, ^73^, ^74^ and such unintentional hypothermia resulting from not monitoring and controlling body temperature might have resulted in reporting an erroneous treatment effectiveness of the treatment. No temperature control was associated with a significantly greater effect size in the xenon studies both with and without CPB. If the xenon treatment resulted in undetected hypothermia, then this could result in an over-estimation of treatment efficacy. However, if poor temperature control results in hypothermia in both control and treatment groups, then the protective effect of hypothermia could mask any protective effect of argon or xenon. Our findings are consistent with the former possibility in studies without temperature measurement.

### Comparison of efficacy of argon and xenon

The main finding of our study is that both argon and xenon have significant positive neuroprotective effect sizes. If we compare argon and xenon in the same three models (CA, TBI, stroke), the difference is pronounced with the effect size of xenon being 34.1% (se, 4.8), a 1.9-fold benefit compared with argon, 18.1% (se, 5.0). Including all the studies we identified, including CPB for xenon, xenon with an effect size of 27.4% (se, 6.3), remained significantly more protective than argon with a 1.5-fold benefit.

### Limitations

Although they are critical to development of new treatments, animal models have many limitations regarding clinical translation, some of which are discussed above. The field of preclinical systematic reviews and meta-analysis is much less well developed than its clinical counterpart. Compared with clinical systematic reviews, there is greater heterogeneity in preclinical meta-analyses owing to differences in the included studies' design, quality, and reporting. In recent years there have been significant improvements in many of these aspects, but it is still uncommon for preclinical experimental protocols to be published in advance, in contrast to clinical trial protocols. This tends to hamper standardisation of experimental protocols between laboratories. There are moves towards publishing preclinical experimental protocols in advance to maximise translational relevance, but these are still in their infancy. In addition, in animal studies of brain injury, it is not straightforward to estimate what a ‘clinically meaningful’ effect size would be (e.g. for reduction in lesion volume). An additional factor is that animal studies use different species. We compared ABI models across three animal species, mouse, rat and pig, that may have differing sensitivities to injury and may manifest the consequences of injury in variable ways. There are valid arguments that larger animal models such as pigs are more representative of human brain injury, particularly as pigs and humans share a gyrencephalic cerebrum, whereas that of rodents is lissencephalic. However, the greater cost and logistical complications of pig models mean that studies are likely to have fewer subjects and to focus on earlier acute outcomes. Rodent models have the advantage of lower cost per animal, an extensive battery of validated behavioural tests, and the possibility of studying chronic effects of ABI on a tractable timescale. It is recognised that, for greater clinical relevance, studies should ideally involve long-term or chronic outcomes. However, until relatively recently most animal ABI studies have been limited to outcomes in the range of days up to a few weeks or months. To include as many studies as possible, we included studies irrespective of time of outcome measurements. Except for one study that used both males and females, all studies used healthy young adult male animals. As a result, no data from females alone, aged animals, or animals with comorbidities were available. Clinically, both males and females experience ABI and there is evidence of differential sensitivity to injury and outcomes.^75^ The older patient community is at particularly high risk for TBI^76^ and stroke,^77^ and older patients usually present with diverse age-related comorbidities, such as hypertension or diabetes. It is important to replicate these neuroprotective effects in hypertensive or diabetic animal groups to improve translation from bench to bedside. A related aspect that may be challenging to model in animals is the polypharmacy associated with comorbidities in older human patients.

Another aspect that we were not able to address in our analysis was the therapeutic time window during which treatment is effective. Only four of the studies we identified have specifically addressed the therapeutic time window with treatment start time as an experimental variable.^38^^,^^42^^,^^45^^,^^56^ In most cases the occurrence of an ABI is unpredictable, and treatment can only be given after injury (CPB and reperfusion after thrombectomy are exceptions). In the case of TBI, treatment before primary injury is not possible, but the aim is to treat promptly before the secondary injury develops significantly. If a treatment is effective only when given before, during, or immediately after the ABI, then it will have limited clinical relevance. Delayed treatment for patients with moderate to severe brain injury may result from long-distance transportation, delayed examination results, shortages of clinicians in the emergency departments, misdiagnosis owing to a lack of specialism, or other circumstances.^78^ Even longer delays often occur in patients with mild brain injury because they may not seek medical help until the symptoms fail to abate several days after injury.^78^ To treat the largest proportion of patients, an appropriate therapeutic time window of at least a few hours with high efficacy maintained is required.

### Clinical relevance

Xenon has already been evaluated clinically in a few early-stage trials: as a treatment for neonatal hypoxic ischaemic brain injury, brain ischaemia after cardiac arrest, and CBP in adults.^70^^,^^79^^,^^80^ The neonatal hypoxic ischaemic brain injury study involved 92 infants and reported no effect on the primary outcomes (lactate levels and MRI fractional anisotropy surrogates of brain injury).^80^ An explanation of this may be that the time of starting xenon treatment, median 10.0 h (inter-quartile range [IQR], 8.2–11.2),^80^ was outside the therapeutic time window (between 3 and 6 h in preclinical studies).^38^ In contrast, an adult out-of-hospital cardiac arrest trial with 110 patients, had a shorter time to xenon treatment (median, 4.1 h; IQR, 3.4–4.6), and reported a positive neuroprotective effect on primary outcome (MRI fractional anisotropy as a surrogate of brain injury).^79^ In CBP in adults, xenon was shown to be safe and feasible, and to reduce S100β, a marker of neuronal injury, but has not progressed to phase 2 efficacy studies.^70^ Our preclinical meta-analysis is equivocal but suggests that xenon may not be effective for this indication, and additional supportive preclinical data of xenon in CPB would be required, particularly in clinically relevant larger animal models. Xenon has not yet been clinically evaluated in stroke or TBI, and our preclinical meta-analysis would support such studies; however, additional preclinical data confirming a clinically useful therapeutic time window would be advisable. Xenon has been reported to increase cerebral blood flow in healthy volunteers^81^ and intracranial pressure (ICP) in patients with TBI,^82^ but other studies have reported no effect on ICP in patients with TBI.^83^^,^^84^ Nevertheless, where an increase in ICP was observed, it is reported that this could be mitigated by hyperventilation.^85^ Given the importance of preventing pathological increases in ICP after TBI, if xenon is used in patients with TBI, it would be important to monitor ICP carefully and mitigate any increases. Future studies on the effect of xenon on ICP after TBI are warranted. At present there have been no human studies of argon as a neuroprotectant in ABI. Our preclinical meta-analysis would support clinical studies of argon in cardiac arrest, stroke, and TBI, although with the caveat that efficacy appears less than that with xenon, and additional data on TBI, including the therapeutic time window, are required.

## Conclusions

Overall, both argon and xenon show neuroprotective effects in the treatment of ABI in animal models, with xenon exerting significantly greater neuroprotective effects than argon. Our findings provide supporting evidence for the application of xenon and argon in clinical ABI therapy, and to guide the design of the future preclinical and clinical study protocols. Additional preclinical studies with both gases to address therapeutic time window and efficacy in female, older, and comorbid animals would be advantageous to facilitate clinical translation.

## Authors' contributions

Study design/planning: RD.

Study conduct: ML, FA, RD.

Data analysis: ML, FA, RD.

Drafting of the paper: RD.

Revision of the paper: ML, FA, RD.
