# Supplementary material for: Neuroprotection by the noble gases argon and xenon as treatments for acquired brain injury: a preclinical systematic review and meta-analysis
Source: Br J Anaesth. 2022 Jun 7;129(2):200–18. doi: 10.1016/j.bja.2022.04.016 (PMC9428918; doi:10.1016/j.bja.2022.04.016)
Supplement: Multimedia component 1 [file mmc1.docx]

**Methods**

**Assessment of study quality – modified CAMARADES score.**

The checklist contained nine risk-of-bias questions including (1) physiological measurements (blood pressure, heart rate, or blood gases), (2) randomisation, (3) blinding of injury protocol, (4) blinding of outcome assessment, (5) sample size calculation, (6) compliance with animal welfare regulations, (7) peer-reviewed publication, (8) potential conflicts of interest and (9) controlling temperature during treatment. For each item, conformity was scored ‘1’, otherwise ‘0’, giving a maximum score of 9. A lower score indicates a higher risk of bias.

Compared to the original CAMARADES checklist we omitted ‘avoidance of an anaesthetic with intrinsic neuroprotective activities’, ^1, 2^ because it is now recognized that all anaesthetics have some neuroprotective effects, ^3, 4^ making this difficult to avoid. Several recent studies have used modified CAMARADES scoring similar to ours that omitted this parameter. ^3, 5, 6^ We also omitted ‘allocation concealment’ because this is often not explicitly reported or is conflated with randomised allocation and judging only according to the description provided might be imprecise and lead to bias. Additionally, ‘injury protocol blinding’ was added to assess whether those carrying out the injury protocol were blinded to the intervention that each animal received during the experiment. ^7^ The field of preclinical systematic reviews and meta-analysis is much less well developed than in the clinical field. Compared to clinical systematic reviews there are fewer validated tools to assess preclinical study methodological quality, but the CAMARADES checklist is one of the most widely used. One of its advantages is that the checklist has an objective binary score for each domain. The relevant domains are either present (1) or not (0) in the experimental design and the publications’ text. Although it cannot assess all aspects of study design and reporting, the CAMARADES checklist is a useful guide to study quality.

**Data extraction & transformation**

When only medians and ranges or interquartile ranges were available, means and SDs were determined using the median, range and sample size. ^8^ When behavioural outcomes were tested more than once at different times, data from each time point was included in the meta-analysis for the paper representing an overall measure of the behavioural outcome for that cohort. ^9^ When studies failed to report data or *n* numbers, the lead or corresponding author of the paper was contacted to retrieve the missing data.

**Calculation of Normalised Mean Difference**

$NMD=100\%\times\frac{\left( \overline{x}_{c}-\overline{x}_{sham} \right)-\left( \overline{x}_{rx}-\overline{x}_{sham} \right)}{\left( \overline{x}_{c}-\overline{x}_{sham} \right)}$ (2)

We used equation (2) to calculate normalized mean difference, where x_c_ is the mean value of control group, x_sham_ is the mean value of sham group and x_rx_ is the mean value of treatment group. ^9^ The standard error in the NMD was calculated as described by Vesterinen *et al*. ^9^ Neurological outcome scores could have increasing values indicating a better outcome or a worse outcome, depending on the scoring system used in the original articles. In cases where neurological outcome was assessed on a scale where larger positive values indicate improved outcome, and there was no sham group, equation (2) was multiplied by -1 to adjust for the direction of effect. ^9^ In preclinical studies where the sample size may be small, random error or experimental model design can result in the observed injury effect (difference between control injury and sham) being small. Hence the denominator in equation (2) may be very small. This can lead to extreme positive or negative NMD values (eg several hundred or thousand percent). We used the method proposed by Vesterinen *et al* ^9^ to deal with cases where the absolute value of effect size calculated using equation (2) is greater than 100% . In these cases the absolute difference between each of the control and treatment groups and the sham group and the effect size is expressed as a proportion of the larger of the two. In cases where ǀ x_c_ - x_sham_ ǀ > ǀ x_rx_ - x_sham_ ǀ we used equation (3); where ǀ x_rx_ - x_sham_ ǀ > ǀ x_c_ - x_sham_ ǀ we used equation 4. Because this calculation of NMD effect size does not account for the direction of effect (e.g. where a higher score represents a better or worse outcome), the sign of the effect size needs to be adjusted by multiplying by -1 if necessary (detrimental effect).

$NMD=100\%\times\frac{\left( \overline{x}_{c}-\overline{x}_{sham} \right)-\left( \overline{x}_{rx}-\overline{x}_{sham} \right)}{\left( \overline{x}_{c}-\overline{x}_{sham} \right)}\times direction$ (3)

$NMD=100\%\times\frac{\left( \overline{x}_{rx}-\overline{x}_{sham} \right)-\left( \overline{x}_{c}-\overline{x}_{sham} \right)}{\left( \overline{x}_{rx}-\overline{x}_{sham} \right)}\times direction$ (4)

The standard deviation of the treatment control group are normalized to the same denominator used to calculate the effect size. Where ǀ x_c_ - x_sham_ ǀ > ǀ x_rx_ - x_sham_ ǀ we used equation (5); where ǀ x_rx_ - x_sham_ ǀ > ǀ x_c_ - x_sham_ ǀ we used equation (6).

${SD}_{c*}=100\%\times\frac{{SD}_{c}}{\left( \overline{x}_{c}-\overline{x}_{sham} \right)}\mathrm{and}{SD}_{rx*}=100\%\times\frac{{SD}_{rx}}{\left( \overline{x}_{c}-\overline{x}_{sham} \right)}$ (5)

${SD}_{c*}=100\%\times\frac{{SD}_{c}}{\left( \overline{x}_{rx}-\overline{x}_{sham} \right)}\mathrm{and}{SD}_{rx*}=100\%\times\frac{{SD}_{rx}}{\left( \overline{x}_{rx}-\overline{x}_{sham} \right)}$ (6)

**Supplementary Table S1. Search strategy**

| #1 | stroke AND (inert gases or xenon or argon) |
| --- | --- |
| #2 | ischemi* AND (inert gases or xenon or argon) |
| #3 | hypoxi* AND (inert gases or xenon or argon) |
| #4 | ischaemi* AND (inert gases or xenon or argon) |
| #5 | #1 OR #2 OR #3 OR #4 |
| #6 | TBI AND (inert gases or xenon or argon) |
| #7 | traumatic brain injury AND (inert gases or xenon or argon) |
| #8 | #6 OR #7 |
| #9 | neuroprot* AND (inert gases or xenon or argon) |
| #10 | neuronal injury AND (inert gases or xenon or argon) |
| #11 | neuronal loss AND (inert gases or xenon or argon) |
| #12 | #9 OR #10 OR #11 |
| #13 | #5 OR #8 OR #12 |
| #14 | argon closed head injury |
| #15 | xenon closed head injury |
| #16 | argon AND neuroprot* AND middle cerebral artery occlusion |
| #17 | xenon AND neuroprot* AND middle cerebral artery occlusion |
| #18 | #14 OR #15 OR #16 OR #17 |
| #19 | #13 OR #18 |
| Full text search phrase (#19) | ((((((((((((stroke AND (inert gases or xenon or argon)) OR (ischemi* AND (inert gases or xenon or argon))) OR (hypoxi* AND (inert gases or xenon or argon))) OR (ischaemi* AND (inert gases or xenon or argon))) OR (TBI AND (inert gases or xenon or argon))) OR (traumatic brain injury AND (inert gases or xenon or argon))) OR (neuroprot* AND (inert gases or xenon or argon))) OR (neuronal injury AND (inert gases or xenon or argon))) OR (neuronal loss AND (inert gases or xenon or argon))) OR (argon AND closed head injury)) OR (xenon AND closed head injury)) OR (argon AND neuroprot* AND middle cerebral artery occlusion)) OR (xenon AND neuroprot* AND middle cerebral artery occlusion) |

**Supplementary Table S2. Modified CAMARADES checklist**

| **Study** | **Mitigation of Bias Score** | | | | | | | | | |
| --- | --- | --- | --- | --- | --- | --- | --- | --- | --- | --- |
|  | **Physiol** | **Random** | **Protocol blinding** | **Assessment blinding** | **Power calculation** | **Regulatory compliance** | **Conflict of interests** | **Peer reviewed publication** | **Control of temperature** | **Total score** |
| **Brücken 2013** | 1 | 1 | 1 | 1 | 0 | 1 | 1 | 1 | 1 | **8** |
| **Brücken 2014** | 1 | 1 | 1 | 1 | 0 | 1 | 1 | 1 | 1 | **8** |
| **Brücken 2015** | 1 | 1 | 1 | 1 | 0 | 1 | 1 | 1 | 1 | **8** |
| **Brücken 2017** | 1 | 1 | 1 | 1 | 1 | 1 | 1 | 1 | 1 | **9** |
| **Creed 2020** | 0 | 1 | 1 | 1 | 1 | 1 | 1 | 1 | 1 | **8** |
| **David 2012** | 1 | 0 | 1 | 0 | 1 | 1 | 1 | 1 | 1 | **7** |
| **Fahlenkamp 2014** | 1 | 1 | 0 | 1 | 0 | 1 | 1 | 1 | 1 | **7** |
| **Fumagalli 2020** | 1 | 1 | 0 | 1 | 1 | 1 | 1 | 1 | 1 | **8** |
| **Liu 2019** | 1 | 1 | 0 | 1 | 1 | 1 | 1 | 1 | 1 | **8** |
| **Ma 2019** | 0 | 1 | 1 | 1 | 0 | 1 | 1 | 1 | 1 | **7** |
| **Moro 2021** | 0 | 1 | 1 | 1 | 0 | 1 | 1 | 1 | 1 | **7** |
| **Ristagno 2014** | 1 | 0 | 0 | 1 | 0 | 1 | 1 | 1 | 1 | **6** |
| **Ryang 2011** | 1 | 1 | 1 | 1 | 1 | 1 | 1 | 1 | 1 | **9** |
| **Zuercher 2016** | 1 | 1 | 0 | 1 | 0 | 1 | 1 | 1 | 1 | **7** |
| **Argon** | **79%** | **86%** | **64%** | **93%** | **42%** | **100%** | **100%** | **100%** | **100%** |  |
|  |  |  |  |  |  |  |  |  |  |  |
| **Campos-Pires 2015** | 1 | 1 | 1 | 1 | 1 | 1 | 1 | 1 | 1 | **9** |
| **Campos-Pires 2019** | 0 | 1 | 1 | 1 | 0 | 1 | 1 | 1 | 1 | **7** |
| **Campos-Pires 2020** | 1 | 1 | 1 | 1 | 1 | 1 | 1 | 1 | 1 | **9** |
| **David 2003** | 1 | 0 | 0 | 1 | 0 | 1 | 0 | 1 | 0 | **4** |
| **David 2008** | 1 | 1 | 0 | 1 | 0 | 1 | 1 | 1 | 0 | **6** |
| **David 2010** | 1 | 1 | 1 | 0 | 0 | 1 | 1 | 1 | 1 | **7** |
| **Derwall 2008** | 1 | 1 | 1 | 0 | 0 | 1 | 1 | 1 | 1 | **7** |
| **Filev 2021** | 0 | 1 | 1 | 1 | 0 | 1 | 1 | 1 | 0 | **6** |
| **Fries 2008** | 1 | 1 | 1 | 1 | 0 | 1 | 1 | 1 | 1 | **8** |
| **Fries 2009** | 1 | 1 | 1 | 1 | 0 | 1 | 1 | 1 | 1 | **8** |
| **Fries 2012** | 1 | 1 | 1 | 1 | 0 | 1 | 1 | 1 | 1 | **8** |
| **Homi 2003** | 1 | 1 | 1 | 1 | 0 | 1 | 1 | 1 | 1 | **8** |
| **Jungwirth 2006** | 1 | 1 | 0 | 1 | 0 | 1 | 0 | 1 | 1 | **6** |
| **Jungwirth 2011** | 0 | 1 | 0 | 1 | 0 | 1 | 1 | 1 | 1 | **6** |
| **Limatola 2010** | 0 | 1 | 0 | 1 | 0 | 1 | 1 | 1 | 0 | **5** |
| **Ma 2003** | 1 | 1 | 0 | 0 | 0 | 1 | 1 | 1 | 1 | **6** |
| **Metaxa 2014** | 1 | 1 | 1 | 1 | 0 | 1 | 1 | 1 | 1 | **8** |
| **Sheng 2012** | 1 | 1 | 1 | 1 | 0 | 1 | 1 | 1 | 1 | **8** |
| **Xenon** | **78%** | **94%** | **67%** | **83%** | **11%** | **100%** | **89%** | **100%** | **78%** |  |
| **Global** | **78%** | **91%** | **65%** | **88%** | **25%** | **100%** | **94%** | **100%** | **88%** |  |

**Supplementary Figure**

**
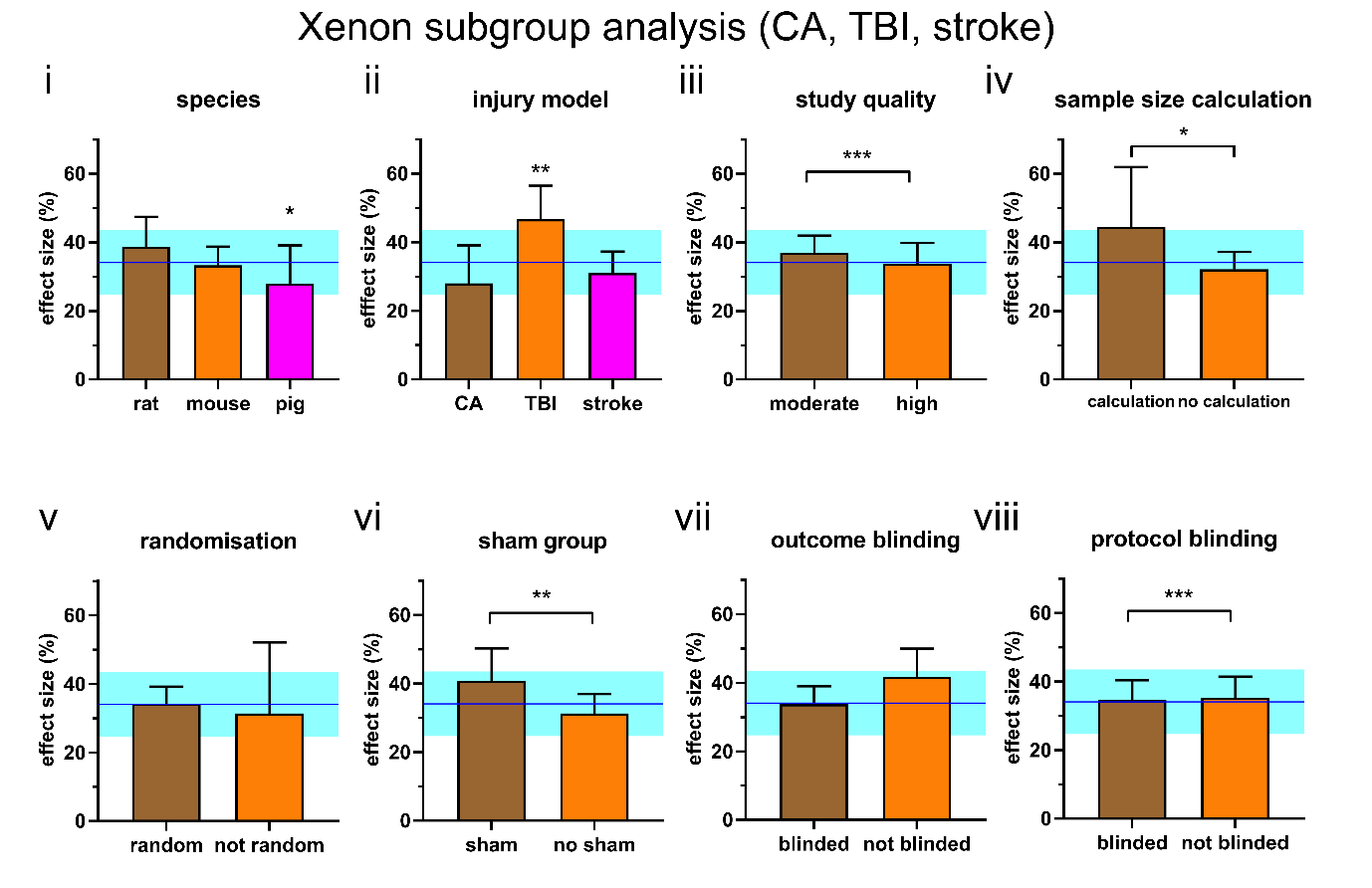
**

**Figure S1**. Neurological outcome effect size comparisons for subgroups in xenon analysis, CA, TBI and stroke models. **(i)** Species, rat (brown bar), mouse (orange bar) and pig (pink bar). **(ii)** Brain injury models, cardiac arrest (CA) (brown bar), traumatic brain injury (orange bar) and stroke (pink bar). **(iii)** Study quality, moderate quality study (score 4-6) (brown bar), *vs* high quality study (score 7-9) (orange bar). **(iv)** Sample size calculation (brown bar) *vs* no sample size calculation (orange bar). **(v)** Randomisation (brown bar) vs no randomisation (orange bar). **(vi)** Sham group (brown bar) vs no sham group (orange bar). **(vii)** Outcome assessment blinded (brown bar) *vs* outcome assessment not blinded (orange bar). **(viii)** injury protocol blinded (brown bar) *vs* injury protocol not blinded (orange bar). Bars are effect size (%), error bars represent SE. Differences between subgroups were tested with chi-square test (* p < 0.05; ** p < 0.01; *** p < 0.001). The overall meta-analysis estimate and 95% CI are indicated by the dark blue line and the light blue shading, respectively.

**Supplementary references**

1 Macleod MR, O'Collins T, Howells DW, Donnan GA. Pooling of animal experimental data reveals influence of study design and publication bias. *Stroke* 2004; **35**: 1203-8

2 Sena E, van der Worp HB, Howells D, Macleod M. How can we improve the pre-clinical development of drugs for stroke? *Trends Neurosci* 2007; **30**: 433-9

3 Archer DP, McCann SK, Walker AM, et al. Neuroprotection by anaesthetics in rodent models of traumatic brain injury: a systematic review and network meta-analysis. *Br J Anaesth* 2018; **121**: 1272-81

4 Archer DP, Walker AM, McCann SK, Moser JJ, Appireddy RM. Anesthetic Neuroprotection in Experimental Stroke in Rodents: A Systematic Review and Meta-analysis. *Anesthesiology* 2017; **126**: 653-65

5 Olai H, Thorneus G, Watson H, et al. Meta-analysis of targeted temperature management in animal models of cardiac arrest. *Intensive Care Med Exp* 2020; **8**: 3

6 Pischiutta F, Caruso E, Lugo A, et al. Systematic review and meta-analysis of preclinical studies testing mesenchymal stromal cells for traumatic brain injury. *NPJ Regen Med* 2021; **6**: 71

7 Hooijmans CR, Rovers MM, de Vries RB, Leenaars M, Ritskes-Hoitinga M, Langendam MW. SYRCLE's risk of bias tool for animal studies. *BMC Med Res Methodol* 2014; **14**: 43

8 Hozo SP, Djulbegovic B, Hozo I. Estimating the mean and variance from the median, range, and the size of a sample. *BMC Med Res Methodol* 2005; **5**: 13

9 Vesterinen HM, Sena ES, Egan KJ, et al. Meta-analysis of data from animal studies: a practical guide. *J Neurosci Methods* 2014; **221**: 92-102
